# Construction of a novel disulfidptosis and cuproptosis-related lncRNA signature for predicting the clinical outcome and immune response in stomach adenocarcinoma

**DOI:** 10.1007/s12672-025-01969-7

**Published:** 2025-02-24

**Authors:** Caihao Qu, Xin Yan, Futian Tang, Yumin Li

**Affiliations:** 1 Lanzhou University Second Hospital, Lanzhou, 730030 China; 2 Key Laboratory of Digestive System Tumors of Gansu Province, Lanzhou, 730030 China

**Keywords:** Disulfidptosis, Cuproptosis, Clinical outcomes, Immune infiltration, NMF, Stomach adenocarcinoma

## Abstract

**Background:**

Disulfidptosis, a newly discovered form of cell death resulting from disulfide stress, remains unclear in its role in stomach adenocarcinoma (STAD). This study aimed to establish a novel disulfidptosis and cuproptosis-related lncRNAs (DCRLs) signature for STAD.

**Methods:**

We sourced RNA-seq data for STAD from the The Cancer Genome Atlas (TCGA) repository. STAD samples underwent nonnegative matrix factorization (NMF) clustering to identify distinct molecular subgroups, followed by Lasso-Cox regression to construct a prognostic model for DCRLs. Subsequently, the model’s clinical predictive capacity was evaluated using a nomogram. The expression of risk lncRNAs was validated via quantitative reverse transcription polymerase chain reaction (qRT-PCR).

**Results:**

The samples were classified into three molecular subtypes based on DCRLs, with the C1 subtype demonstrating the worst prognosis. We identified four independent prognostic lncRNAs (AC016394.2, NUTM2A-AS1, OIP5-AS1, and LIMS1-AS1) and constructed a prognostic risk model. Survival analysis revealed that high-risk patients had a poorer prognosis. The model’s risk score was strongly correlated with the tumor mutational burden (TMB), microsatellite instability (MSI), immune subtypes, and tumor-infiltrating immune cells (TIICs) in the tumor microenvironment (TME). Analysis utilizing the Tumor Immune Dysfunction and Exclusion (TIDE) revealed a higher risk of tumor immune evasion among high-risk patients. Moreover, the expression levels of four risk lncRNAs were higher in the majority of gastric cancer cell lines compared to normal cell lines.

**Conclusion:**

Our study establishes a risk model that effectively predicts clinical outcomes and immune response in STAD.

**Supplementary Information:**

The online version contains supplementary material available at 10.1007/s12672-025-01969-7.

## Introduction

Gastric cancer (GC) is a prevalent malignant tumor that carries significant morbidity and mortality rates, being ranked 5th and 3rd, respectively, worldwide [1]. Among all GC histological types, stomach adenocarcinoma (STAD) accounts for over 90% and exhibits notable characteristics such as fast development, strong invasion, and high malignancy [2]. Unfortunately, the majority of people with GC are identified at advanced stages, forfeiting their best opportunity to receive surgical treatments, which results in a five-year survival rate of under 20% [3]. Under such circumstances, building a reliable prognostic model that offers a new perspective for diagnosing and treating STAD is extremely urgent.

In February 2023, Liu et al. [4] first proposed a novel form of cell death referred to as “disulfidptosis”. Their research indicated that cells with high SLC7A11 expression generated disulfide stress due to the accumulation of intracellular disulfides such as cystine under glucose starvation conditions. This resulted in the formation of an increased number of disulfide bonds in the actin-regulated cytoskeleton, which caused significant contraction and detachment from the cell membrane, disrupting the cytoskeletal structure and ultimately bringing about cell death [4]. In March 2022, Tsvetkov et al. [5] identified a new copper-dependent form of cell death known as “cuproptosis”. This mode of cell death was found to be mediated by protein lipidation and associated with mitochondrial activity. Studies have shown a significant increase in copper levels in cancer patients, and greater levels may promote cancer cell proliferation, metastasis, and tumor angiogenesis [5, 6]. Disulfiram (DSF), acts as an ion carrier, delivering Cu^2+^ to cells and causing intracellular copper overload and severe oxidative damage. Moreover, DSF is metabolized in vivo to diethyl dithiocarbamate (DDC), which forms Cu (DDC)_2_ complexes that chelate with intratumoral Cu^2+^ and selectively induce apoptosis in cancer cells [7]. Studies have found that disulfidptosis is associated with poor prognosis in glioma and lung adenocarcinoma patients [8, 9]. Cuproptosis and cuproptosis-related lncRNAs have been consistently linked to an unfavorable prognosis in patients with hepatocellular carcinoma [10, 11].

Long noncoding RNAs (lncRNAs) are more than 200 nucleotides in length, comprising a group of genetically heterogeneous gene transcripts and enhancer RNAs [12]. Through histone modification and post-transcriptional regulation, lncRNA is capable of modulating the expression, translation, and stability of coding genes [13]. A growing body of research indicates that lncRNAs are essential to the development and spread of cancer, affecting critical cellular processes such as cell proliferation, invasion, and metastasis [14].

Currently, little research has looked at the predictive potential of disulfidptosis and cuproptosis-related lncRNAs (DCRLs) in STAD. In this work, we built a predictive model based on DCRLs using the TCGA. Meanwhile, we explored and analyzed the role of the model in the immune response, immune escape, and chemotherapy sensitivity. The expression of risk lncRNA in STAD was verified by qRT-PCR. We believe that our research can provide a preliminary reference for the selection of treatment options for patients with STAD.

## Materials and methods

### Data collection

RNA-Seq and clinical information on STAD were acquired from the TCGA database (https://portal.gdc.cancer.gov/, accessed on January 2023) [15], encompassing 412 STAD and 36 normal gastric tissue samples. The RNA-Seq expression profiles were divided into mRNA-Seq matrix and lncRNA-Seq matrix with Perl (perl-5.30.0.1) software.

### Extraction of DCRLs

We obtained disulfidptosis and cuproptosis genes from a literature review [4, 5, 28–31], subjecting them to Pearson analysis to screen DCRLs with |R > 0.3| and *P* < 0.01. Furthermore, Sankey diagrams were drawn using R packages “dplyr”, “ggalluvian”, and “ggplot2” to visualize DCRLs. Shared lncRNAs were obtained using Venn diagram intersection and mRNA-lncRNA network maps were drawn using the Cytoscape tool. The R packages “org.Hs.eg.db” and “enrichplot” were used for enrichment analysis of disulfidptosis and cuproptosis genes.

### NMF classification of DCRLs subgroups

NMF clustering analysis was executed on the DCRLs to establish distinct molecular subtypes. The “NMF” package and the “brunet” method, along with the cophenetic, residuals, and sparseness indicators, were utilized to identify the optimal number of clusters. Based on the analysis, the optimal number of clusters was determined to be 3.

### Construction of the DCRLs prognostic signature

The TCGA-STAD dataset was subjected to a random 1:1 ratio division into a training set and a test set. Upon the training set, we formulated a DCRLs prediction model and subsequently evaluated its reliability utilizing the test dataset and total set. LncRNAs related to prognosis were screened using the “survival” package and Coxph function. Furthermore, we adopted Lasso regression and multivariable Cox analysis to establish the risk prognosis model, where we calculated the risk score for each STAD patient as Risk score = ∑ (coefficient_i_ × expression value of lncRNA_i_).

### Evaluation of efficacy and clinical value of prognostic prediction model

The risk score’s median value was used to classify the STAD samples into high-risk and low-risk groups. We employed Kaplan–Meier survival analysis and Cox regression to assess the risk score’s OS and prognostic independence, respectively. Additionally, we constructed a nomogram model for forecasting the 1-, 3-, and 5-year OS rates of patients with STAD. The calibration curve, ROC curve, and C-index were used to evaluate the model’s predictive efficacy and clinical predictive value.

### Principal component analysis (PCA) and immune response

We plotted the PCA for stratifying patients in high-risk and low-risk groups using the “scatterplot3d” package. Tumor immune rejection and immune escape are analyzed using the TIDE database (http://tide.dfci.harvard.edu/, accessed on January 2023) [32]. Additionally, we identified five immune subtypes in TCGA-STAD, namely C1: wound healing, C2: IFN-γ dominant, C3: inflammatory, C4: lymphocyte depleted, and C6: TGF-β dominant.

### Tumor microenvironment

To quantify immune cell infiltration and immune-related function activation in patients in high-risk and low-risk groups, we performed single sample Gene Set Enrichment Analysis (ssGSEA) on STAD samples using the “Gene Set Variation Analysis (GSVA)” and “GSEAbase” R packages. Moreover, we employed the “estimate” package to determine the TME score for every STAD patient. The abundance of tumor-infiltrating immune cells (TIICs) was quantified using CIBERSORT in STAD samples, and the connection between the risk score and TIICs was examined using the Spearman test.

### Chemotherapeutic agents

We utilized the “oncoPredict” R package to determine the half maximal inhibitory concentration (IC50) for each STAD sample, with the sensitivity of chemotherapeutic drugs to patients in the high and low-risk groups analyzed to identify potentially effective agents for STAD treatment. The 3D molecular structure of the drug was displayed via the PubChem database (https://pubchem.ncbi.nlm.nih.gov/) [16].

### qRT-PCR

Human gastric cancer cell lines AGS-1, MKN-28, MKN-45, HGC-27, NCI-N87, and human normal gastric mucosal cells GES-1 were acquired from the Cell Resource Center (Beijing, China). The cells were cultured in RPMI-1640 (Gibco, USA) medium and maintained at 37 °C in a 5% CO_2_ incubator. Total RNA was extracted from the cells TRIzol reagent (Invitrogen, USA) and reverse transcribed into cDNA using cDNA Synthesis Kit (Accurate Biology, AG, China). Fluorescence quantitative PCR reaction was conducted with the following PCR reaction conditions: initial denaturation at 95 °C for 30 s, followed by 40 cycles of amplification at 95 °C for 5 s and 60 °C for 30 s. The primer sequence of lncRNAs was displayed in Supplemental Table 1. Relative lncRNA expression was calculated using the 2^−△△Ct^ method.

### Statistical

In this study, statistical analysis and graphical visualization were carried out using R software (version 4.1.3). A log-rank test was used to compare survival disparities. A *P* < 0.05 was considered statistically significant.

## Results

### Screening of DCRLs

The flowchart is showed in Supplemental Fig. 1. We obtained 10 disulfidptosis genes and 19 cuproptosis genes. Co-expression analysis of the mRNA matrix with the lncRNA matrix identified DCRLs (Supplemental Fig. 2A, B). Through the Venn diagram, we obtained 138 shared lncRNAs for disulfidptosis and cuproptosis (Supplemental Fig. 2C). Network analysis of the disulfidptosis and cuproptosis genes with shared lncRNAs was carried out using the Cytoscape tool (Supplemental Fig. 2D). Additionally, enrichment analysis was carried out for disulfidptosis and cuproptosis genes. GO analysis revealed that they are enriched in the sulfur compound metabolic process, mitochondrial matrix, and oxidoreductase activity (Supplemental Fig. 2E, Supplemental Table 2), and KEGG analysis showed that they are enriched in the TCA cycle and metabolic pathway (Supplemental Fig. 2F, Supplemental Table 3).

**Fig. 1:**
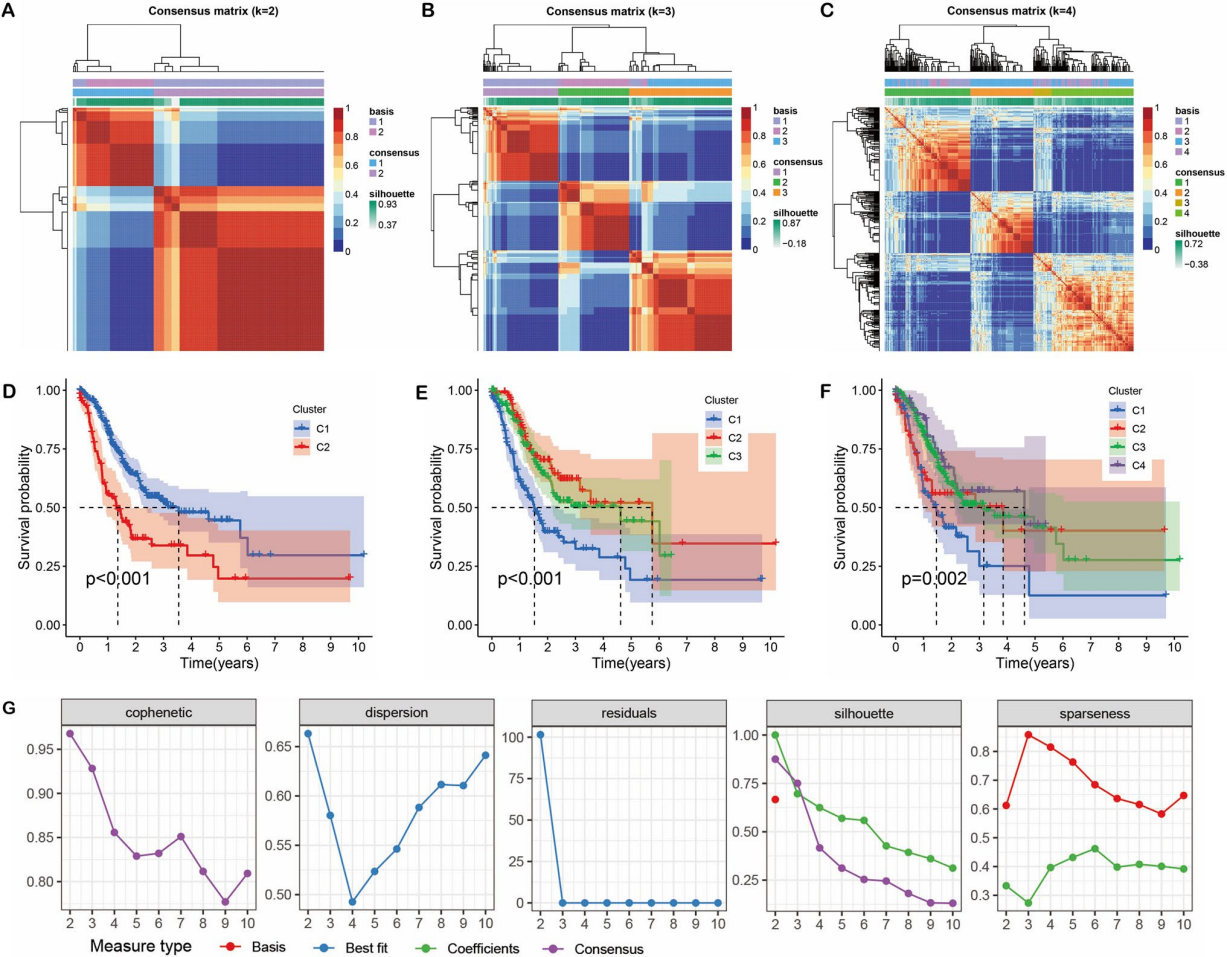
NMF of DCRLs subgroups. **A–C** Clustering heatmaps were generated for coefficient values k = 2, 3, and 4. **D–F** Survival curves were generated for coefficient values k = 2, 3, and 4. **G** Measure type assessment of DCRLs

**Fig. 2:**
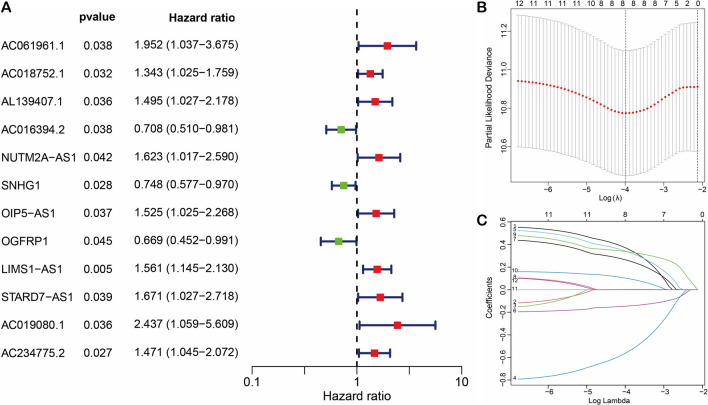
Cox-Lasso regression analysis of DCRLs. **A** Univariate Cox regression analysis. **B–C** Lasso regression analysis

### Nonnegative matrix factorization (NMF) of DCRLs subgroups

The molecular subgroup of 138 DCRLs was conducted using NMF clustering. Subsequently, clustering heatmaps were generated for coefficient values k = 2, 3, and 4 (Fig. 1A–C). Analysis of survival curves revealed variations among the cluster types associated with different coefficient values (Fig. 1D–F). Furthermore, through Measure type assessment, it was determined that the optimal number of clusters is k = 3 (Fig. 1G).

### Construction of risk prediction models based on DCRLs

To identify the prognosis of DCRLs in STAD patients, we randomly divided them into training and test sets. Univariate Cox analysis of the training set enabled us to identify 12 prognosis-related lncRNAs (Fig. 2A), while Lasso regression further helped us in screening 8 prognosis-significant lncRNAs (Fig. 2B, C). Multivariate Cox regression analysis identified 4 independent prognostic lncRNAs (AC016394.2, NUTM2A-AS1, OIP5-AS1, and LIMS1-AS1) and constructed a prognostic model for STAD. The risk score of each STAD patient was calculated using the following formula = (−0.8385099 × AC016394.2exp) + (0.43234768 × NUTM2A-AS1exp) + (0.50719108 × OIP5-AS1exp) + (0.41934268 × LIMS1-AS1exp).

**Fig. 3:**
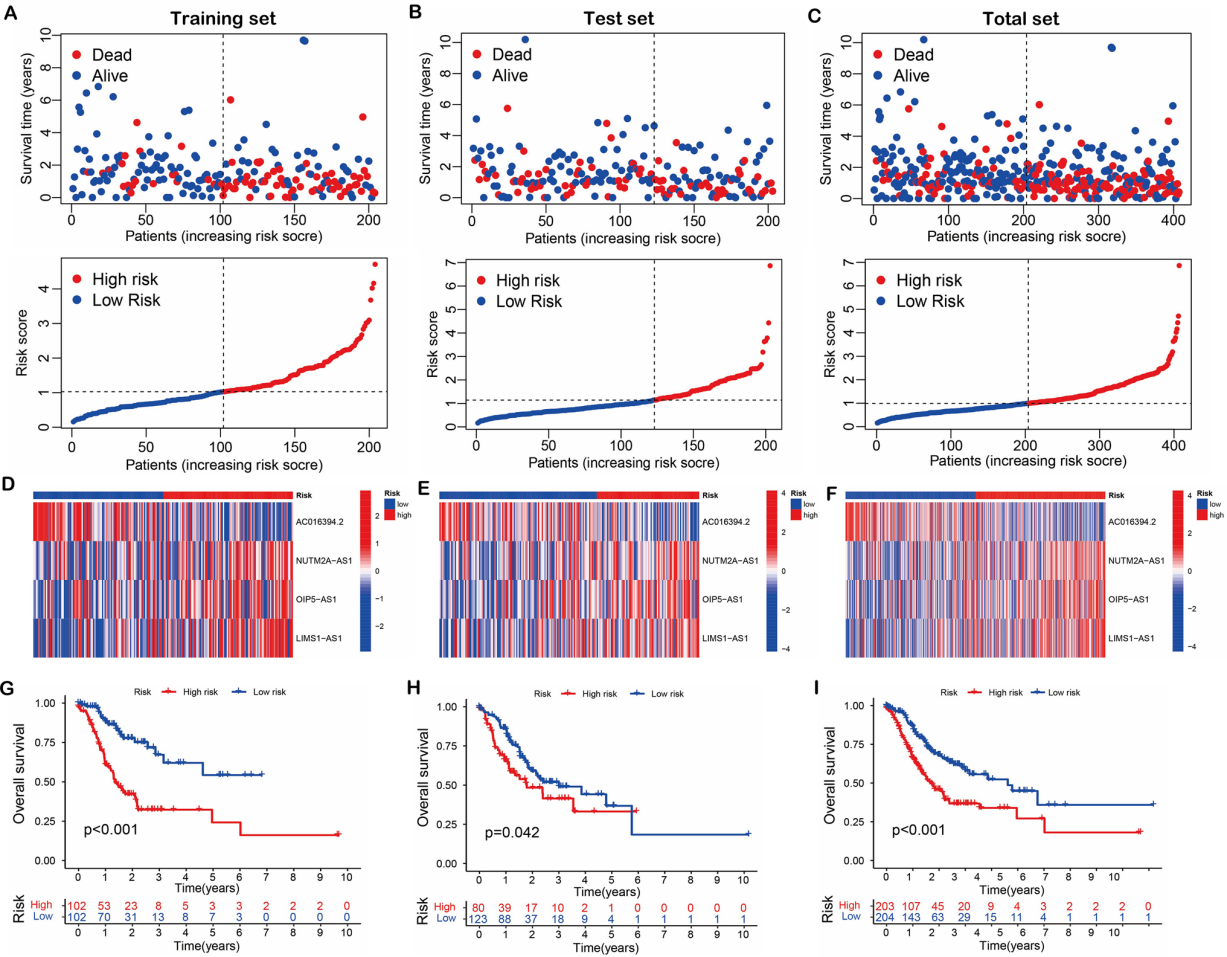
Risk scores of the training set, test set and total set. **A–C** Survival state plots of risk scores for the three datasets. **D–F** heatmaps of risk scores for the three datasets. **G–I** Kaplan–Meier survival curves of risk scores for the three datasets

To examine the reliability of our prognostic model, we generated survival status plots, heatmaps, and Kaplan–Meier curves for STAD patients in the training set, test set, and total set, respectively. The survival status plots verified that patients in the high-risk groups had poor survival times in the three data sets (Fig. 3A–C). Moreover, the heatmap showed that risk lncRNA expression was higher in the high-risk groups in the three datasets (Fig. 3D–F). Consistent with these findings, Kaplan–Meier survival curves revealed that patients in the high-risk groups had significantly lower overall survival (OS) rates compared to the low-risk groups in the three data sets (Fig. 3G–I).

### Prediction efficiency and clinical value of risk prognosis model

**Fig. 4:**
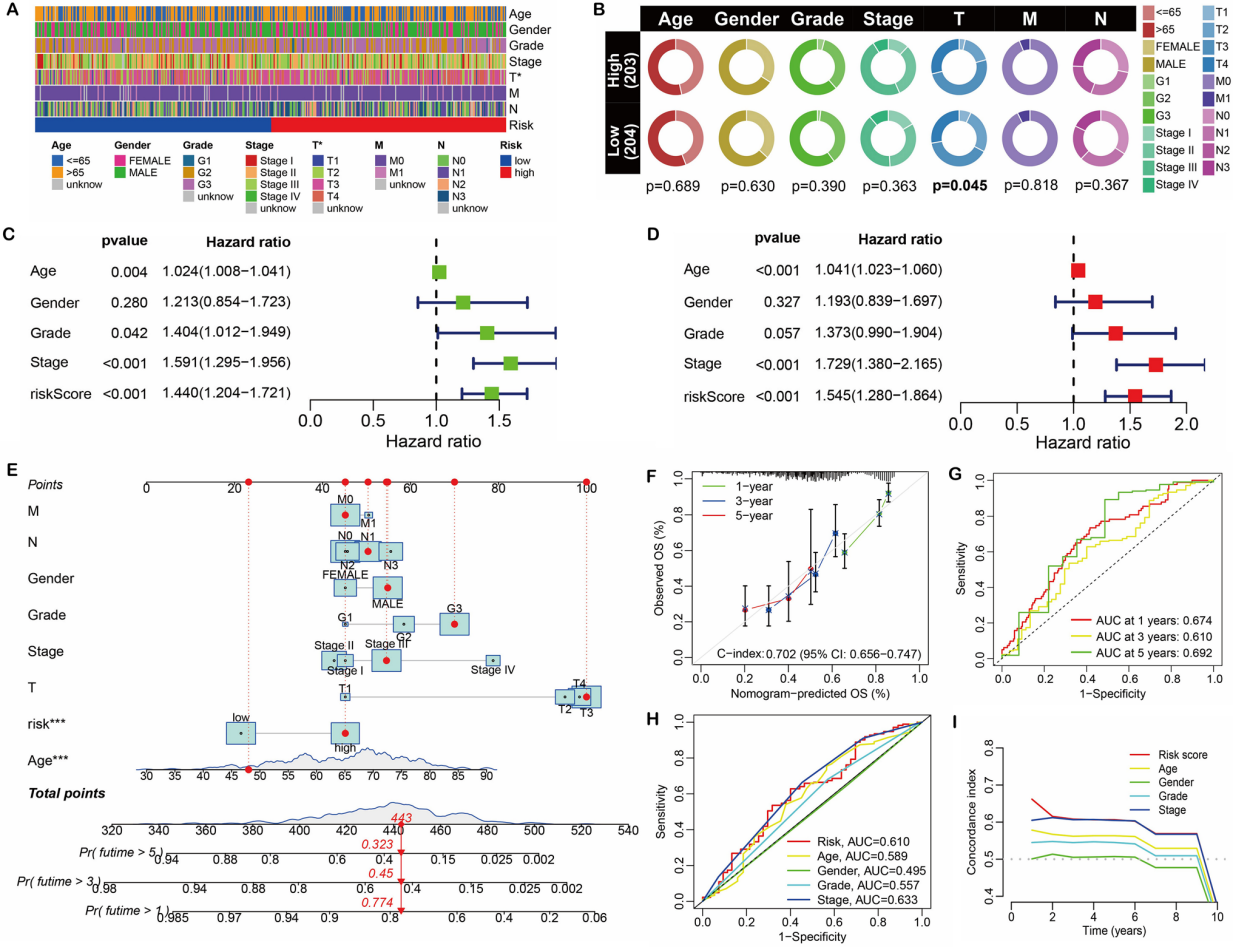
Prognostic efficacy and clinical value of prognostic model. **A–B** Relationship between risk score and clinical data. **C–D** Univariate and multivariate Cox analysis of risk scores. **E** Nomogram based on risk score. **F** Calibration curve of nomogram. **G** ROC curve of nomogram. H ROC curves of risk scores and clinical factors. **I** C-index of risk scores and clinical factors

In this research, we found a link between risk score and T stage (Fig. 4A, B). Furthermore, the risk score was also shown to be an independent prognostic factor using Cox regression analysis (Fig. 4C, D). The risk score was used to create the nomogram model (Fig. 4E), and the model’s C-index was 0.702 (95% CI: 0.656–0.747) (Fig. 4F). Notably, the calibration curve demonstrates that the predicted OS and the observed OS are both consistent (Fig. 4F). The nomogram model’s AUC values at 1-, 3-, and 5-years OS were 0.674, 0.610, and 0.692, respectively. (Fig. 4G). Additionally, we calculated the AUC value and C-index of the factors in the model and discovered that the risk score was a better prognostic indicator (Fig. 4H, I).

### GSVA (gene set variation analysis) and immune checkpoints (ICPs)

**Fig. 5:**
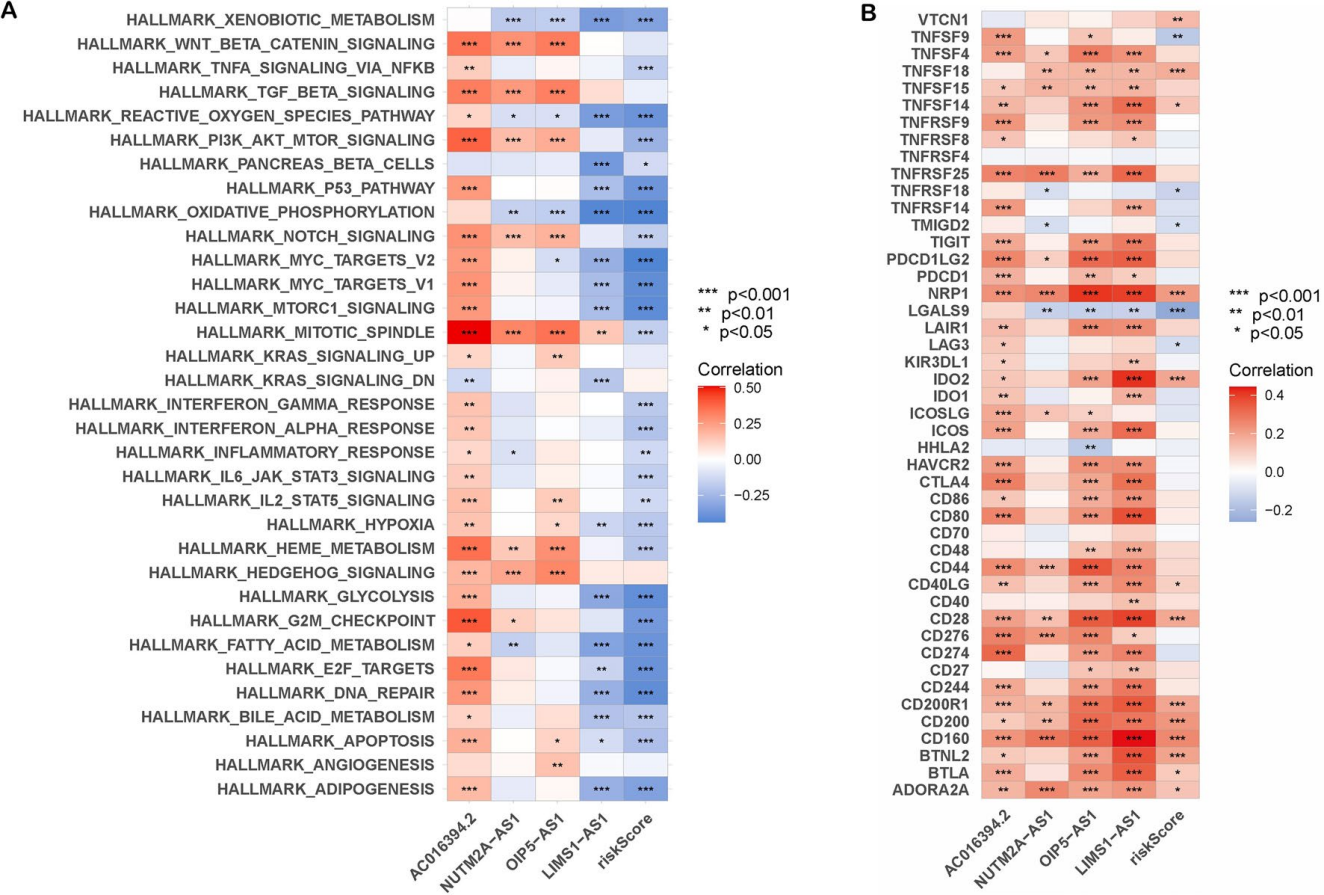
GSVA pathways and ICPs. **A** The correlation between risk score and GSVA pathway. **B** The correlation between risk score and immune checkpoints

Through GSVA enrichment analysis, it was determined that the risk score exhibited strong associations with NOTCH signaling, IL6 JAK STAT3 signaling, and P53 signaling (Fig. 5A). In addition, it was found that the risk score was significantly correlated with NRP1, CD28, CD200 and other immune checkpoints (Fig. 5B).

### PCA and immune response

**Fig. 6:**
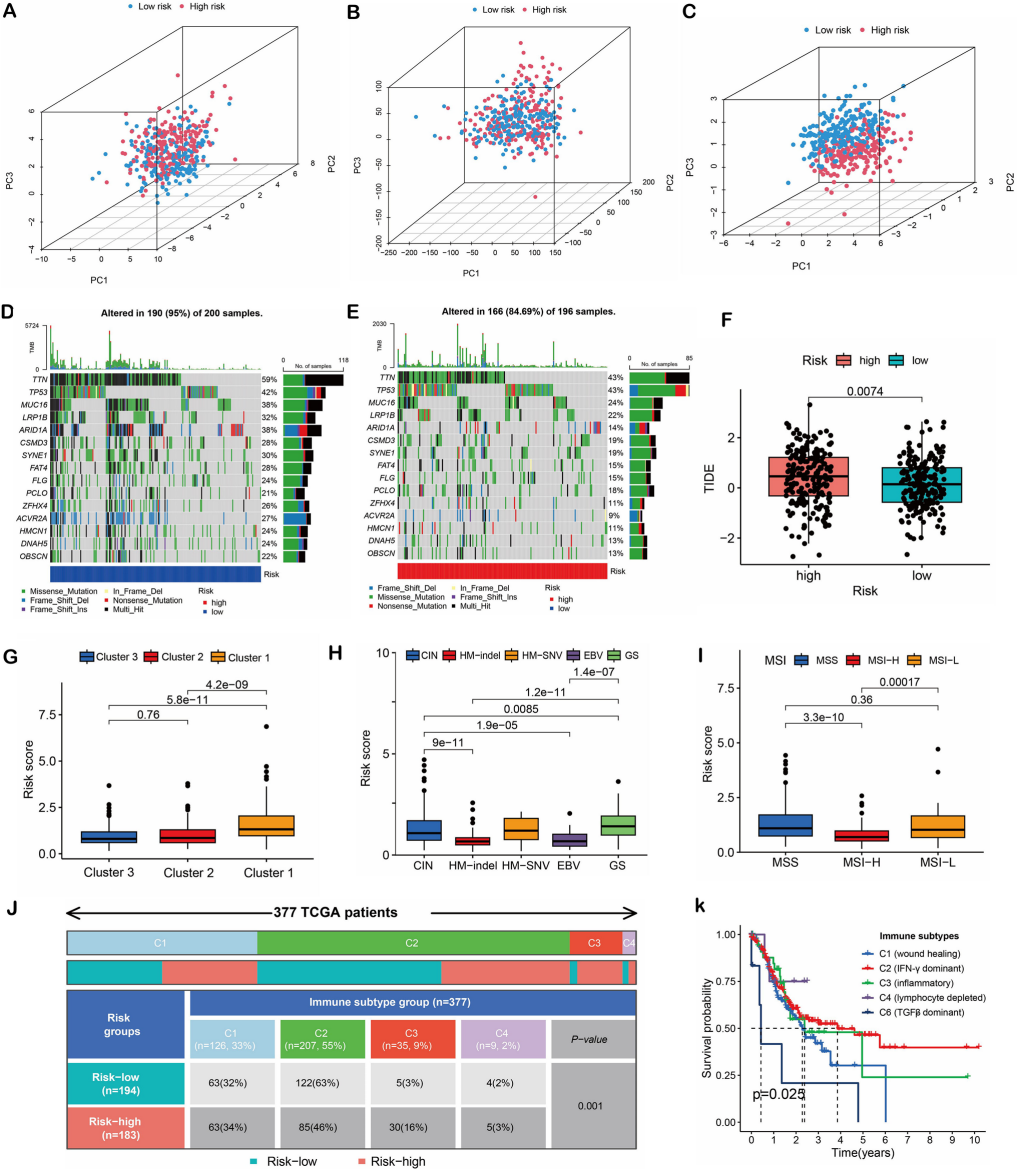
PCA and immune response. **A** Disulfidptosis and cuproptosis genes profiles of PCA. **B** The shared lncRNAs of PCA. **C** The model risk lncRNAs of PCA. **D** Mutation spectrum of low-risk groups. **E** Mutation spectrum of high-risk groups. F Immune escape of risk scores was analyzed using the TIDE database. **G** Risk score of NMF clustering. **H–I** Molecular subtypes. **J** Immune subtypes. **K** Survival analysis of immune subtypes

We conducted a PCA on disulfidptosis and cuproptosis genes, shared lncRNAs, and risk lncRNAs to compare the differences between high-risk and low-risk groups (Fig. 6A–C). Our results revealed that the prognostic risk model was good at separating patients into high and low-risk groups. To more thoroughly research the underlying molecular pathways, we examined the mutation profiles of patients in both risk groups. Our results revealed that although the genes mutated in the high-risk and low-risk groups were nearly the same, the specific mutation types were different. For instance, within the low-risk group, the TTN gene mainly showed Multi_Hit mutation, followed by Missense_mutation; conversely, this trend was reversed in the high-risk group (Fig. 6D, E). Furthermore, we assessed immune dysfunction, immune exclusion, and immune escape in STAD using the TIDE database to evaluate the efficacy of immune checkpoint inhibitors (ICIs) in patients. We found that the low-risk group had lower TIDE scores, indicating better prediction of response to ICIs treatment (Fig. 6F). Risk scores differed among the cluster subtypes (Fig. 6G). Moreover, we observed differences between risk scores and molecular subtypes (EBV, GS, HM, CIN, and MSI) (Fig. 6H, I). Furthermore, notable distinctions were observed in immune subtypes (C1, C2, C3, C4, and C6) among the high and low-risk groups (Fig. 6J). Notably, survival analysis indicated that patients with C6 subtype demonstrated the most unfavorable prognosis (Fig. 6K). Hence, the model’s risk score exhibited a favorable immune response in STAD.

### Tumor microenvironment

**Fig. 7:**
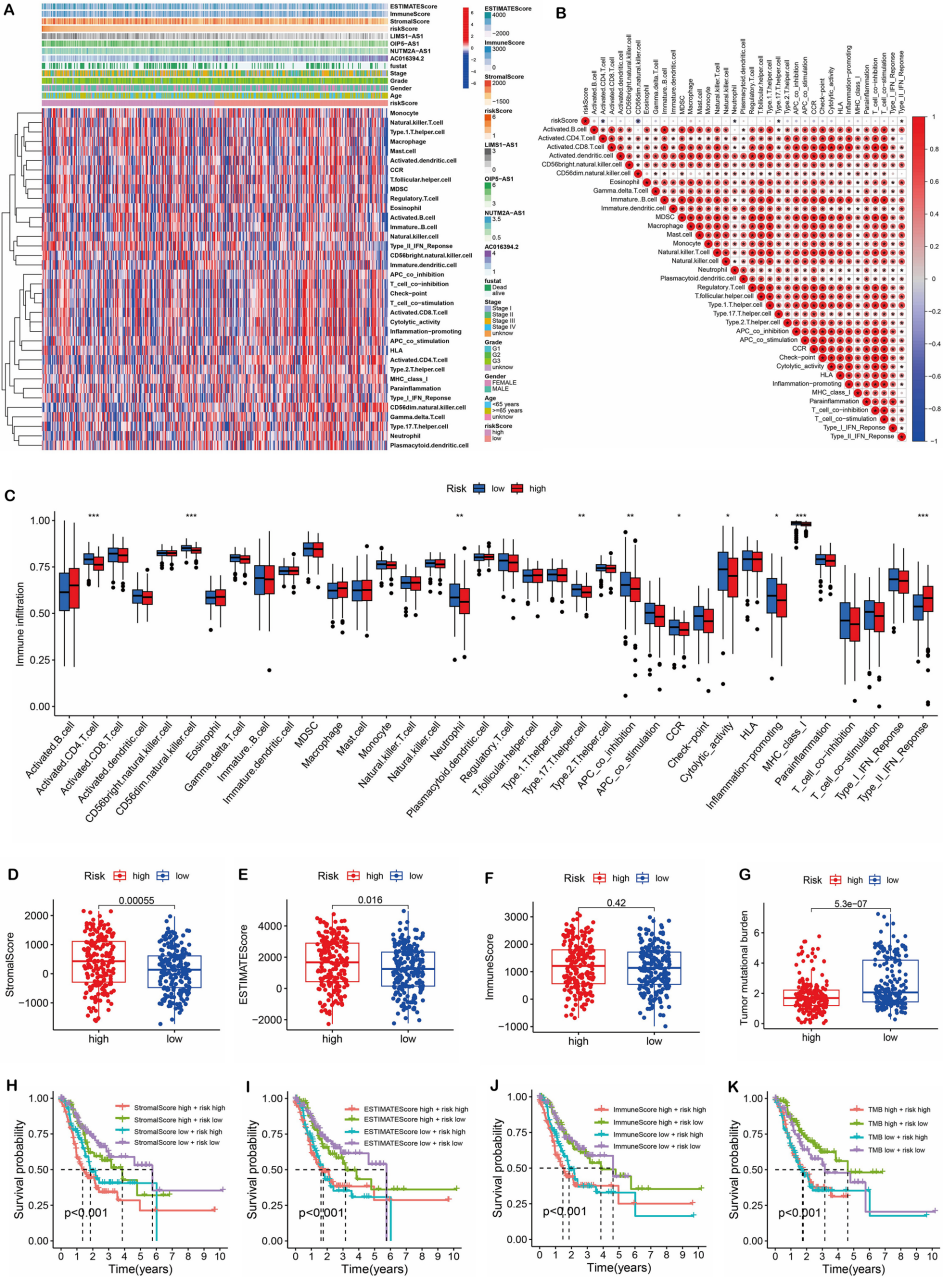
Tumor microenvironment. **A** Heatmap of the relationship between risk score and TME. **B** Correlation of immune cells with each other. **C** Correlation between risk score and immune cells. **D–G** Differences in Stromal scores, ESTIMATE scores, Immune scores and TMB scores between high and low risk score groups. **H–K** Survival analysis of Stromal scores, ESTIMATE scores, Immune scores and TMB scores in combination with risk scores. *P < 0.05, **P < 0.01, ***P < 0.001

We utilized ssGSEA to explore the role of risk scores in the TME of STAD. Heatmaps of immune cells, immune functions, TME scores, and risk lncRNAs were generated, as depicted in Fig. 7A. The association between immune cells and risk score was investigated using correlation analysis (Fig. 7B). Our findings demonstrated that levels of immune infiltration of activated CD4 T cell, Neutrophil, and APC_co_inhibition were higher in the low-risk group, whereas levels of immune infiltration of Type_II_IFN responses were higher in the high-risk group (Fig. 7C). Furthermore, we evaluated the Stromal score, ESTIMATE score, and TMB in high-risk and low-risk groups of STAD patients. We found that the Stromal score and ESTIMATE score were higher in the high-risk groups, while TMB was higher in the low-risk groups (Fig. 7D–G). To determine the prognostic value of these factors, we conducted a survival analysis, revealing significant survival differences between risk score and Stromal score, ESTIMATE score, Immune score, and TMB (Fig. 7H–K).

**Fig. 8:**
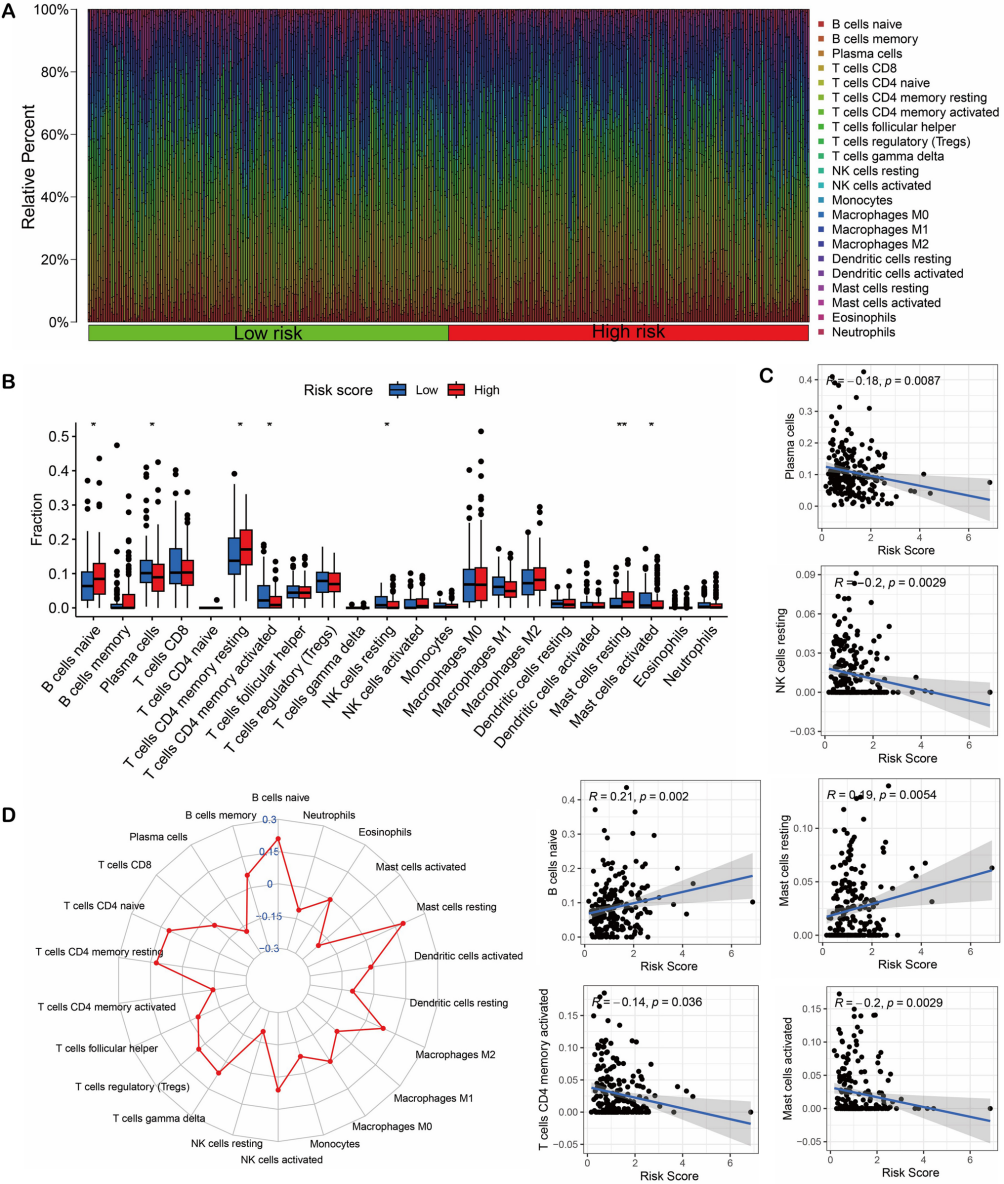
Relationship between TIICs and risk scores. **A** The content of TIICs in the STAD sample. **B** Differences in TIICs between high and low-risk score groups. **C** Radar plot of the relationship between TIICs and risk scores. **D** Correlation analysis of TIICs and risk scores. *P < 0.05, **P < 0.01

To further investigate the TME, we quantified the infiltration abundance of TIICs in STAD samples using the CIBERSORT algorithm. The percentage of TIICs in each STAD sample was illustrated with a heatmap (Fig. 8A). Subsequent analysis revealed that B cells naive, T cells CD4 memory activated, and Mast cells activated remarkably differed between high-risk and low-risk groups (Fig. 8B). Additionally, we utilized radar plots to demonstrate the correlation between risk scores and TIICs, revealing a strong correlation between risk scores and B cells naive, and Mast cells activated (Fig. 8C, D).

### Individualized precision medicine

**Fig. 9:**
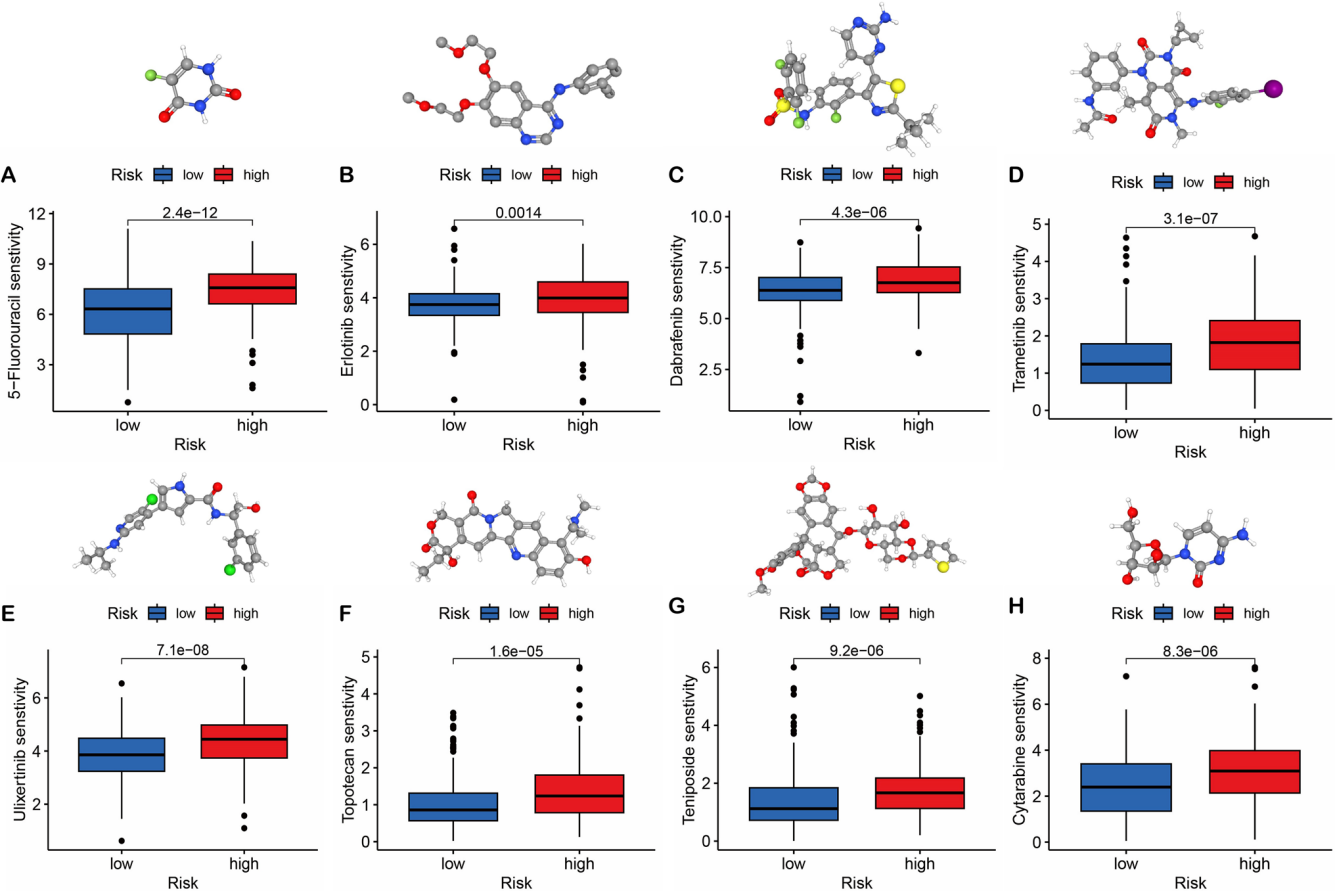
The sensitivity and 3D molecular structures of chemotherapeutic agents. **A** 5-Fluorouracil. **B** Erlotinib. **C** Dabrafenib. **D** Trametinib. **E** Ulixertinib. **F** Topotecan. **G** Teniposide. **H** Cytarabine

The sensitivity and 3D molecular structures of chemotherapeutic agents were examined using the oncoPredict algorithm and PubChem database, and it was discovered that high-risk patients can achieve better therapeutic effects with 5-Fluorouracil, Erlotinib, Dabrafenib, Trametinib, Ulixertinib, Topotecan, Teniposide, Cytarabine (Fig. 9A–H).

### The expression of risk lncRNAs in STAD was verified by qRT-PCR

**Fig. 10:**
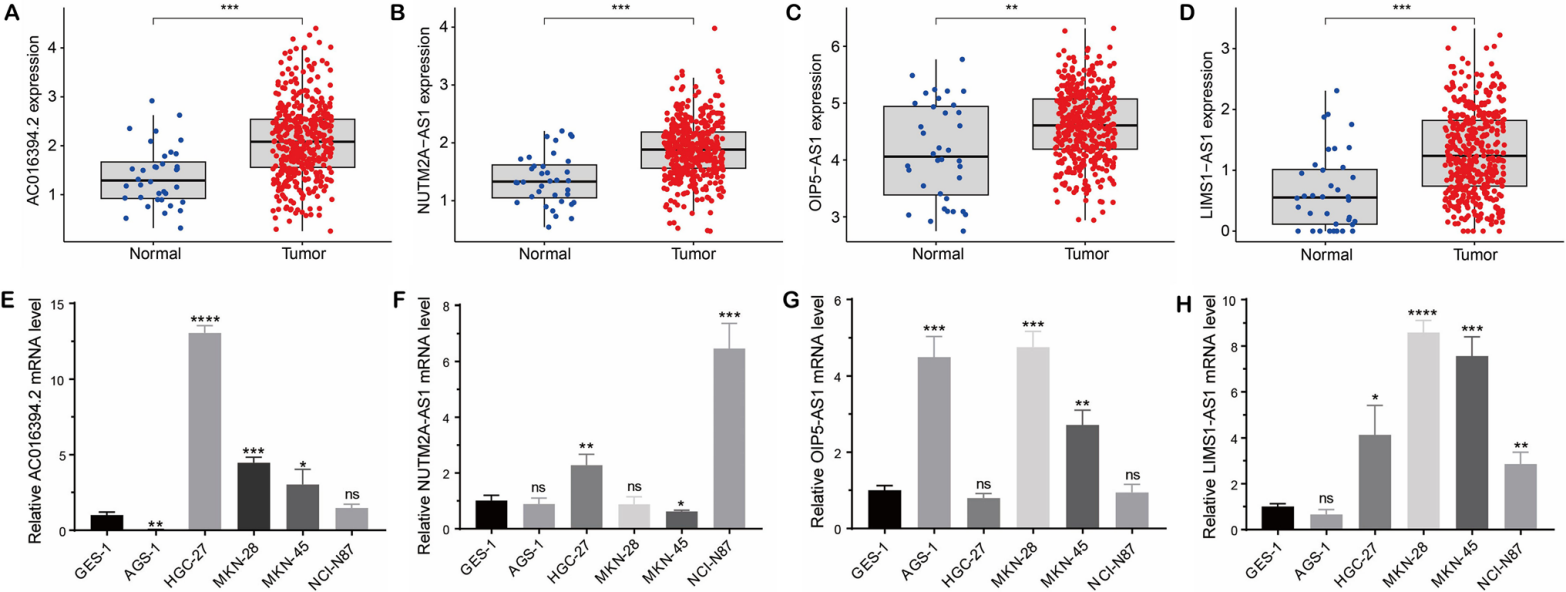
The expression level of risk lncRNAs in STAD. **A–D** The expression levels of AC016394.2, NUTM2A-AS1, OIP5-AS1, and LIMS1-AS1 in both normal and cancer tissues. **E–H** Relative expression levels of AC016394.2, NUTM2A-AS1, OIP5-AS1, and LIMS1-AS1 in normal cell lines and GC cell lines via qRT-PCR. *P < 0.05, **P < 0.01, ***P < 0.001, ****P < 0.0001, ns *P* > 0.05

The expression levels of four risk lncRNAs (AC016394.2, NUTM2A-AS1, OIP5-AS1, and LIMS1-AS1) were evaluated in 36 normal tissue samples and 412 tumor tissue samples. Our analysis revealed that all four lncRNAs were significantly upregulated in cancerous tissues (Fig. 10A–D). Furthermore, the expression levels of four risk lncRNAs were higher in majority of GC cell lines compared to normal cell lines (Fig. 10E–H).

## Discussion

Disulfidptosis is a novel type of cell death that occurs under glucose-deficient conditions. Unlike other forms of programmed cell death, disulfidptosis results from disulfide stress due to the accumulation of excess cystine in cells [4, 17, 18]. Cuproptosis caused the loss of iron-sulfur cluster proteins and the aggregation of lipid-acylated proteins in the tricarboxylic acid cycle, which ultimately led to proteotoxic stress response and cell death [5, 19]. Under in vitro conditions, DSF generates highly oxidized DDC intermediates. The process of DDC chelation with Cu^2+^ also involves redox reactions, resulting in the generation of reactive oxygen species (ROS) [20]. NF-κB is a redox-sensitive transcription factor that exhibits potent antiapoptotic activity. It has been established that DSF/Cu treatment stimulates the inactivation of the NF-kB pathway and activation of the ROS-JNK pro-apoptotic pathway, ultimately triggering apoptosis [20]. Extensive research has demonstrated the critical role of lncRNAs in influencing the occurrence, progression, and prognosis of various types of tumors, including GC [21]. Furthermore, lncRNAs have been recognized as essential regulators of immune responses and immune cell function in GC [22, 23]. As such, exploring lncRNA as a potential therapeutic target could offer promising avenues for the treatment of STAD.

Due to the complexity of the molecular features of STAD, a variety of potential lncRNAs biomarkers can be used to comprehensively evaluate the prognosis of patients. In this study, we focused on disulfidptosis and cuproptosis, two recent cell death modes, and constructed a shared lncRNA prognostic model. Our model was better able to distinguish high and low-risk populations and showed significant survival differences and ideal predictive value. Additionally, we observed differences in immune response to the TME and sensitivity to drug therapy between high-risk and low-risk groups, suggesting that our model provides a theoretical basis for making subsequent clinical treatment and prognosis decisions for individualized patients.

In this study, a total of 4 lncRNAs (AC016394.2, NUTM2A-AS1, OIP5-AS1, and LIMS1-AS1) were included to construct a clinical prognostic model. Tu et al. [24] identified that AC016394.2 has independent prognostic value in GC. NUTM2A-AS1 has been implicated in promoting hepatocellular carcinoma by activating the Wnt/β-catenin pathway [25]. OIP5-AS1 has been shown to promote cell proliferation and aerobic glycolysis in GC [26]. STAD patients were divided into low-risk and high-risk groups using the risk score formula. Survival analysis indicated that high-risk patients had worse outcomes, and Cox regression identified risk scores as an independent prognostic factor. Moreover, heatmap and PCA further demonstrated the ability of this model to differentiate between risk groups. Taken together, a nomogram model based on risk score was constructed to more accurately predict individual survival rates for STAD patients, providing a theoretical basis for more personalized clinical treatment decisions.

The TME has an essential role in promoting malignant biological behavior and chemoresistance in tumors [27]. Immune cells are a crucial aspect of the TME and possess potential prognostic value [28, 29]. In this research, the Stromal score, ESTIMATE score, immune functions, and immune cells in the TME were different between the high-risk and low-risk groups. CD4 + T cells play a crucial role in tumor immunity and therapy. They enhance the immune system’s ability to attack tumor cells by assisting other immune cells, producing cytokines, forming immune memories, and differentiating into different functional subsets [30]. Neutrophils can secrete cytokines to enhance the anti-tumor immunity of the body and attract other immune cells to gather around the tumor to form infiltration and play its killing role [31]. Our study found that patients in the low-risk group had higher levels of activated CD4 T cells and neutrophils, which predicted a better prognosis in the low-risk group. Therefore, the results suggested that the risk score influenced the recruitment of immune infiltrating cells and the modulation of immune function in STAD. This provides a new basis for immunotherapy strategies for patients with STAD. TMB is a critical predictor of the efficacy of immunotherapy for tumors [32]. Patients with higher TMB tend to have better outcomes and may respond well to ICIs and chemotherapy [33]. MSI was a marker for effective ICI treatment, and GC patients with MSI-H had a better prognosis [34]. This study found that patients in the MSI-H group had a lower risk score, indicating that low-risk patients had a better prognosis and were more suitable for immunotherapy. In addition, TMB was higher in the low-risk group than in the high-risk group, which also predicted better outcomes and better response to immunotherapy in low-risk patients. The TIDE scores were also lower in the low-risk groups, indicating that the patients in these groups responded better to the ICI therapy. Thus, this suggests that patients in low-risk groups benefit more from ICI treatment. In addition, risk scores were closely related to immune subtypes (C1-C4, C6), and molecular subtypes (MSI, EBV, GS, CIN). Using pharmacogenomics analysis, it was predicted that 5-fluorouracil and cytarabine may be effective drugs for patients in the high-risk group. Chen et al. [35] revealed that 5-fluorouracil inhibited proliferation and promoted apoptosis in GC cells. Cytarabine is a chemotherapeutic agent commonly used in clinical practice for postoperative chemotherapy of GC, demonstrating significant therapeutic efficacy [36]. These results provide a valuable reference for individualized precision therapy and inspire the development of new drug options for STAD patients.

Nevertheless, the study is not exempt from limitations. First, it is an exploratory study based on bioinformatics analysis, which lacks the additional validation of large clinical samples. Second, although we identified the prognostic role of these lncRNAs in STAD patients, the underlying mechanisms as relevant prognostic markers remain unclear, thus warranting further extensive validation studies.

## Conclusions

In conclusion, our study established a clinical predictive prognostic model based on disulfidptosis and cuproptosis-related lncRNA, which provides a certain reference value for the individualized precision treatment and short-term follow-up of STAD patients. In addition, the model’s risk score has good predictive performance for the clinical outcome and immune response of STAD.

## Supplementary Information

Additional file 1

Additional file 2

Additional file 3

## Data Availability

The RNA-seq raw read count, Transcripts Per Kilobase of exon model per Million mapped reads (TPM) and the related clinical data from patients with STAD were extracted from The Cancer Genome Atlas (TCGA, https://portal.gdc.cancer.gov/projects/TCGA-STAD/) database which were used for the construction of our signature. The datasets generated and/or analysed during the current study are available in the TCGA, https://portal.gdc.cancer.gov/projects/TCGA-STAD/.
