# Supplementary material for: Construction of a novel disulfidptosis and cuproptosis-related lncRNA signature for predicting the clinical outcome and immune response in stomach adenocarcinoma
Source: Discov Oncol. 2025 Feb 24;16:230. doi: 10.1007/s12672-025-01969-7 (PMC11850681; doi:10.1007/s12672-025-01969-7)
Supplement: Supplementary file 2 — Additional file 2 [file 12672_2025_1969_MOESM2_ESM.docx]

**Supplemental Table**

**Supplemental Table 1 The primer sequence of lncRNAs.**

| LncRNA | Primer sequence |
| --- | --- |
| AC016394.2 | Forward: CCGAGCAACATCACTTCCCT |
| AC016394.2 | Reverse: AATGAGGTGTGGCGAGTCAG |
| NUTM2A-AS1 | Forward: CCCCATCTGTGTCTTTCGCT |
| NUTM2A-AS1 | Reverse: AAGGGTAAGGGAGCAGGTCA |
| OIP5-AS1 | Forward: GAGACCACCAAACAGGCTTT |
| OIP5-AS1 | Reverse: AGTGAAGTTTCAGCGGTGGT |
| LIMS1-AS1 | Forward: ACACAGCTGCTTCCTTTCTGT |
| LIMS1-AS1  GAPDH  GAPDH | Reverse: GCATGGTGCTGGCTGATAAC  Forward: GGAGCGAGATCCCTCCAAAAT  Reverse: GGCTGTTGTCATACTTCTCATGG |

**Supplemental Table 2 GO enrichment analysis of disulfidptosis and cuproptosis-related genes.**

| GO | Description | P-value | FDR |
| --- | --- | --- | --- |
| BP:0006790 | sulfur compound metabolic process | 3.26E-11 | 2.76E-08 |
| BP:0006086 | acetyl-CoA biosynthetic process from pyruvate | 1.31E-09 | 3.65E-07 |
| BP:0006084 | acetyl-CoA metabolic process | 1.61E-09 | 3.65E-07 |
| BP:0044272 | sulfur compound biosynthetic process | 1.72E-09 | 3.65E-07 |
| BP:0006637 | acyl-CoA metabolic process | 5.33E-09 | 7.53E-07 |
| CC:0005759 | mitochondrial matrix | 3.24E-17 | 2.98E-15 |
| CC:1990204 | oxidoreductase complex | 4.36E-14 | 2.01E-12 |
| CC:0045239 | tricarboxylic acid cycle enzyme complex | 8.31E-07 | 2.55E-05 |
| CC:0098798 | mitochondrial protein-containing complex | 4.23E-05 | 0.000973 |
| CC:0042470 | melanosome | 0.000554 | 0.008499 |
| MF:0016620 | NAD or NADP as acceptor | 3.19E-07 | 3.38E-05 |
| MF:0016903 | oxidoreductase activity | 6.51E-07 | 3.45E-05 |
| MF:0016747 | acyltransferase activity, amino-acyl groups | 1.10E-06 | 3.88E-05 |
| MF:0016746 | acyltransferase activity | 2.19E-06 | 5.80E-05 |
| MF:0051536 | iron-sulfur cluster binding | 3.80E-06 | 6.72E-05 |

**Supplemental Table 3 KEGG enrichment analysis of disulfidptosis and cuproptosis-related genes.**

| KEGG | Description | P-value | FDR |
| --- | --- | --- | --- |
| hsa00020 | Citrate cycle (TCA cycle) | 2.23E-08 | 1.54E-06 |
| hsa01200 | Carbon metabolism | 9.94E-07 | 3.43E-05 |
| hsa01100 | Metabolic pathways | 1.84E-06 | 4.22E-05 |
| hsa00620 | Pyruvate metabolism | 4.99E-06 | 8.60E-05 |
| hsa00010 | Glycolysis / Gluconeogenesis | 4.65E-05 | 0.000642275 |
| hsa01524 | Platinum drug resistance | 6.16E-05 | 0.000708122 |
| hsa05230 | Central carbon metabolism in cancer | 0.001126057 | 0.011099707 |
| hsa00630 | Glyoxylate and dicarboxylate metabolism | 0.003640582 | 0.029535031 |
| hsa04922 | Glucagon signaling pathway | 0.003852395 | 0.029535031 |
| hsa00640 | Propanoate metabolism | 0.004660521 | 0.032157597 |
| hsa00260 | Glycine, serine and threonine metabolism | 0.006408477 | 0.036848741 |
| hsa04216 | Ferroptosis | 0.006408477 | 0.036848741 |
| hsa00380 | Tryptophan metabolism | 0.007047912 | 0.037408149 |
