# Supplementary material for: Construction of a novel disulfidptosis and cuproptosis-related lncRNA signature for predicting the clinical outcome and immune response in stomach adenocarcinoma
Source: Discov Oncol. 2025 Feb 24;16:230. doi: 10.1007/s12672-025-01969-7 (PMC11850681; doi:10.1007/s12672-025-01969-7)
Supplement: Supplementary file 3 — Additional file 3 [file 12672_2025_1969_MOESM3_ESM.docx]

**Supplementary Figure**

**
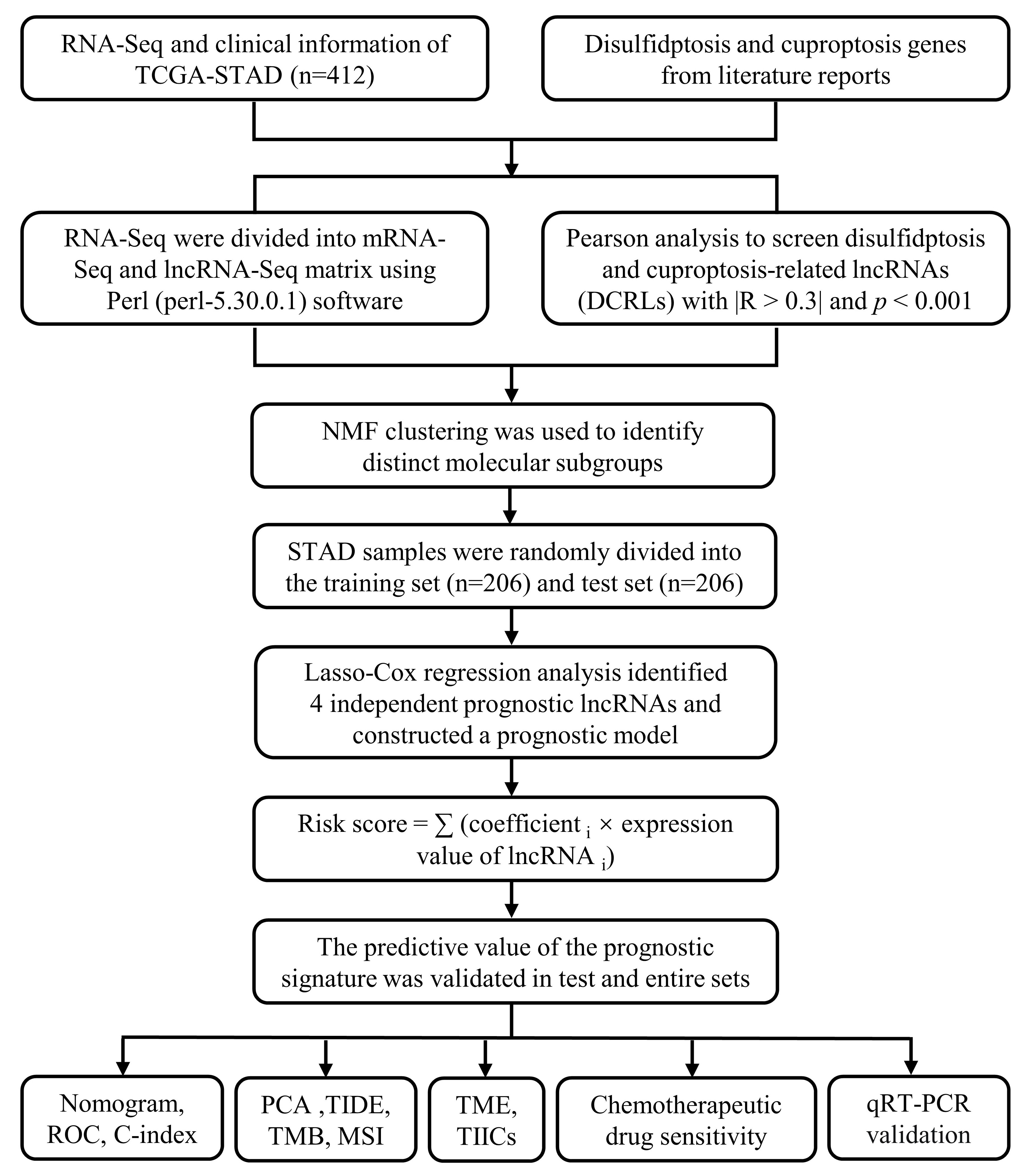
**

**Supplementary Figure 1:** The work flowchart of the study. Abbreviations: TCGA: The Cancer Genome Atlas, NMF: nonnegative matrix factorization, ROC: receiver operator characteristic, PCA: principal component analysis, TIDE: tumor immune dysfunction and exclusion, TMB: tumor mutational burden, MSI: microsatellite instability, TME: tumor microenvironment, TIICs: Tumor infiltrating immune cells.


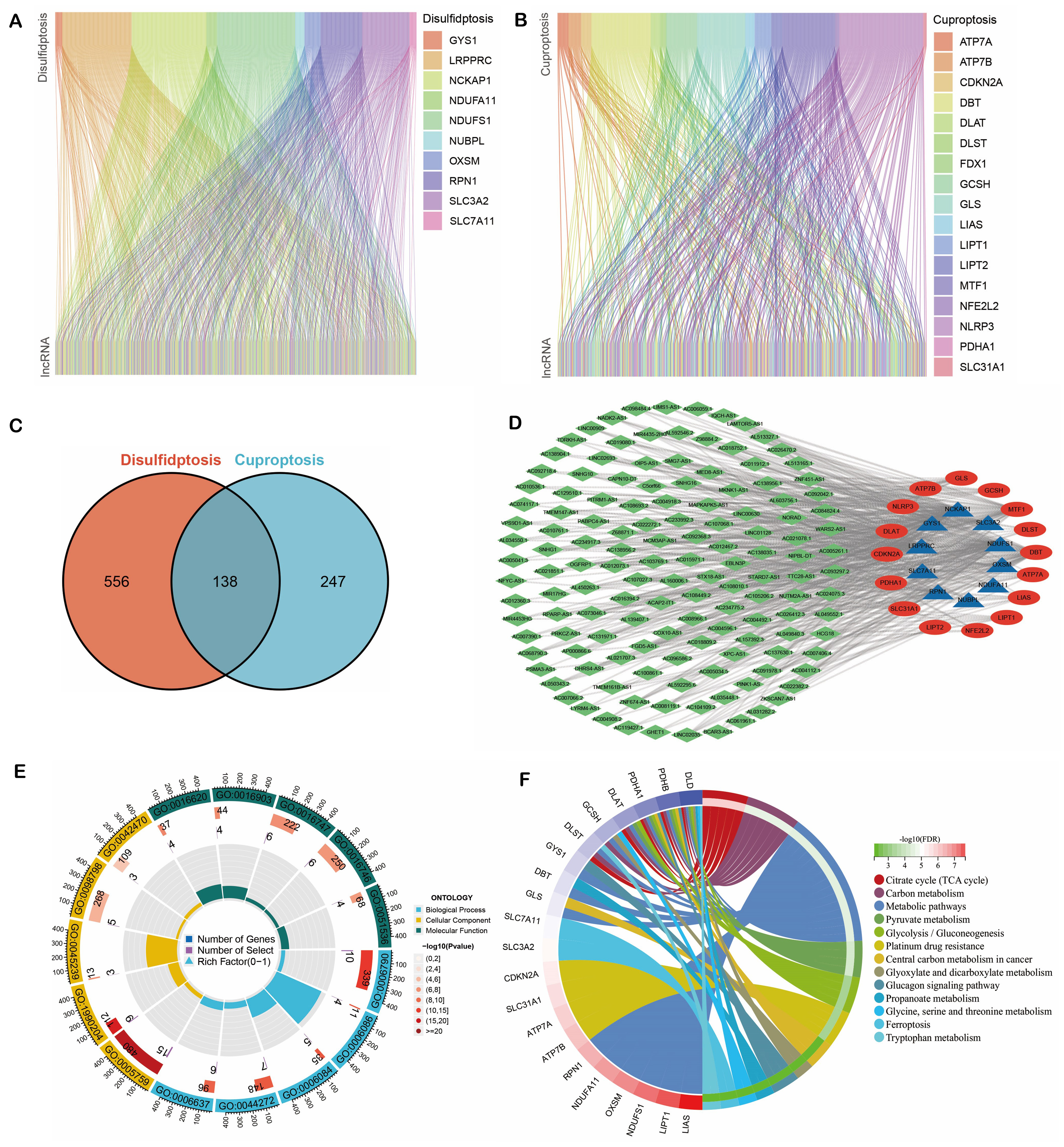


**Supplementary Figure 2:** Disulfidptosis and cuproptosis-related lncRNAs (DCRLs). (A) Disulfidptosis-related lncRNAs. (B) Cuproptosis-related lncRNAs. (C) Shared lncRNAs for disulfidptosis and cuproptosis. (D) Network diagram of disulfidptosis and cuproptosis genes with shared lncRNAs. (E) GO enrichment analysis of disulfidptosis and cuproptosis genes. (F) KEGG enrichment analysis of disulfidptosis and cuproptosis genes.
